# The COVID-19 infection control arms race

**DOI:** 10.1017/ice.2020.211

**Published:** 2020-05-08

**Authors:** Chanu Rhee, Meghan A. Baker, Michael Klompas

**Affiliations:** 1 Department of Population Medicine, Harvard Medical School and Harvard Pilgrim Health Care Institute, Boston, Massachusetts; 2 Division of Infectious Diseases, Brigham and Women’s Hospital, Boston, Massachusetts

## Abstract

US hospitals are engaged in an infection control arms race. Hospitals, specialties, and professional groups are spurring one another on to adopt progressively more aggressive measures in response to COVID-19 that often exceed federal and international standards. Examples include universal masking of providers and patients; decreasing thresholds to test asymptomatic patients; using face shields and N95 respirators regardless of symptoms and test results; novel additions to the list of aerosol-generating procedures; and more comprehensive personal protective equipment including hair, shoe, and leg covers. Here, we review the factors underlying this arms race, including fears about personal safety, ongoing uncertainty around how SARS-CoV-2 is transmitted, confusion about what constitutes an aerosol-generating procedure, increasing recognition of the importance of asymptomatic infection, and the limited accuracy of diagnostic tests. We consider the detrimental effects of a maximal infection control approach and the research studies that are needed to eventually de-escalate hospitals and to inform more evidence-based and measured strategies.

US hospitals are engaged in an infection control arms race. Hospitals, specialties, and professional groups are spurring one another on to adopt progressively more aggressive infection control measures that often exceed the core standards set by the Centers for Disease Control and Prevention (CDC) and the World Health Organization (WHO). Hospitals are caught in a cycle wherein whenever one hospital moves to new standard practice that is perceived as more protective, another feels intense pressure to follow. Professional societies accelerate the cycle by making unilateral proclamations about expected standards for their members. As soon as one hospital agrees to the new standard, providers at other institutions point to these examples as de facto evidence that their hospital must follow. Examples include universal masking of providers and patients; decreasing thresholds to test asymptomatic patients; using face shields and N95 respirators regardless of symptoms and test results; novel additions to the list of aerosol-generating procedures; and more comprehensive personal protective equipment including hair, shoe, and leg covers.

The infection control arms race is driven, understandably, by fear. We are all alarmed by the news of countless COVID-19–related deaths; the case fatality rate is at least 10 times that of seasonal influenza.^1^ Perhaps more terrifying is the fact that many of the patients who are dying are young and healthy, and many are healthcare workers. In China, 4% of confirmed COVID-19 cases in the first month occurred among medical staff, and even higher rates have been reported in Europe.^2^ In many cases, these infections were due to delayed recognition of COVID-19 rather than PPE failures, but the impression has nonetheless taken hold that healthcare workers using standard PPE are not safe.

Conflicting and changing recommendations from federal and international authorities have goaded the arms race by sowing doubt in providers’ minds. In February, the WHO recommended contact and droplet precautions (ie, gown, gloves, medical masks, and eye protection) for most COVID-19 patients while reserving N95 respirators or powered air-purifying respirators (PAPRs) for patients undergoing aerosol-generating procedures.^3^ The CDC initially recommended N95 respirators for all COVID-19 patients but shifted to allowing medical masks in times of N95 shortages. This shift gives the impression that CDC guidance is driven by supply shortages rather than science and that medical masks are inferior to N95 respirators. This concern is further exacerbated by scattered reports raising the possibility that SARS-CoV-2 may be carried in aerosols, although none of these have yet demonstrated aerosol-based transmission.^4-6^

The arms race is further fueled by the realization that anyone may be carrying the virus. Several studies have now documented that presymptomatic patients are contagious and have high viral burdens.^7-9^ But there is a tendency to conflate the estimated prevalence of asymptomatic infection among patients with confirmed infections, thought to range between 20% and 50%, with the estimated prevalence of asymptomatic infection in the general population, which appears to be closer to 1%–2% in most areas.^8,10-12^ These findings compel providers to want to test all patients and to use maximal precautions regardless of symptoms and epidemiological risk factors. Even negative tests are not trusted following reports that the sensitivity of a single nasopharyngeal polymerase chain reaction (PCR) test may be as low as 70% and that a nonnegligible number of confirmed cases initially tested negative.^13,14^ Indeed, the CDC recently updated their guidance to recommend that hospitals in areas with high community prevalence of COVID-19 consider using N95 respirators in all asymptomatic patients undergoing aerosol-generating procedures regardless of SARS-CoV-2 testing results.^15^

Hospitals are now faced with threading the needle between allaying providers’ fears, responding to shifting guidance from public health authorities and declarations from professional societies, and managing pressing equipment shortages. One of the most contentious issues is deciding which procedures are “aerosol generating” and therefore warrant N95 respirators. Unfortunately, there are no universally accepted criteria. Intubation, bronchoscopy, cardiopulmonary resuscitation, nebulization, and noninvasive positive-pressure ventilation have been associated with respiratory virus transmissions, but little or no compelling data have documented respiratory virus transmission for most other procedures.^16^ Nonetheless, an increasing number of professional societies are creating their own definitions of aerosol-generating procedures based on theoretical concerns rather than documented transmissions.^17-20^ These procedures now include endoscopies, laparoscopies, nasogastric tube placements, labor and delivery, and any other procedure that has the potential to induce coughing. However, coughing is a cardinal symptom of COVID-19 even in the absence of a procedure, so it is difficult to understand how a procedure that merely induces coughing is meaningfully different from routine care of a COVID-19 patient.

With the global shortage of N95s, most hospitals do not have the luxury of being able to deploy N95s for every patient interaction or even for procedures that some providers consider aerosol generating. We believe that hospital administrators’ first obligation is to assure that adequate PPE will always be available for providers performing the highest risk procedures in the highest risk patients, but in so doing they must withhold N95s from others and therein raise the ire of these providers who feel unsafe and undervalued. These feelings are further validated in the minds of providers when hospitals’ policies run contrary to the recommendations of their professional societies or do not match the standards provided by other hospitals.

Beyond N95 respirators, some providers are now wearing head coverings, shoe coverings, and leg coverings. These enhancements to standard PPE are motivated in some cases by PPE standards in other countries as well as reports documenting high rates of environmental contamination in the rooms of some COVID-19 patients.^6,21^ Providers are rarely reassured by the fact that is no convincing evidence to date of viral transmission from shoes, hair, or pant bottoms or that isolation of SARS-CoV-2 in the environment by PCR does not necessarily equate to viability. Some providers are also requesting PAPRs even when adequately fit-tested to N95 respirators. Anecdotes circulate about anesthesiologists or surgeons refusing to intubate or perform surgery on asymptomatic patients without N95 respirators and preoperative testing.

In light of these trends, it is only a matter of time before hospitals converge on a uniform, maximal infection control strategy. We fear this will include N95 respirators, face-shields, gown, and gloves for all patient encounters regardless of symptoms, and likely even more PPE for symptomatic patients undergoing aerosol-generating procedures. Symptomatic patients will be tested multiple times using PCRs followed by serological tests if PCR testing is negative. Furthermore, all patients will be tested for COVID-19 upon hospital admission and serially thereafter. Patients undergoing elective surgeries and procedures will also be tested for COVID-19 once if not twice, even if negative test results do not allay providers’ desire to use full PPE including N95 respirators. To decrease the risk of healthcare exposures from staff, more hospitals will likely begin regularly screening providers with COVID-19 tests and checking serologies. The timetable for hospitals to reach this point will vary by access to resources, local competition, provider pressure, and local COVID-19 prevalence (and indeed, some hospitals in the hardest hit areas have already adopted many of these strategies), but it increasingly seems that this will be the end game for most hospitals around the country.

Can—and should—anything be done to de-escalate this arms race? There are clear down sides to this maximal infection control and testing approach. COVID-19 test kits are in short supply, and every additional patient placed on respiratory isolation precautions contributes to a hospital’s dwindling supplies of gowns, gloves, medical masks, face shields, goggles, N95 respirators, PAPRs, disinfectant wipes, and alcohol-based hand sanitizers. Donning full PPE can lead to delays in delivering emergent care such as cardiopulmonary resuscitation and intubations. Even for routine care in the prepandemic era, prior studies have suggested that contact precautions are associated with less patient–healthcare worker contact, decreased patient satisfaction, increased depression and anxiety, and other adverse consequences.^22^ The unnecessary use of negative-pressure rooms for surgical procedures in patients undergoing aerosol-generating procedures may also increase the risk of surgical site infection.

At the same time, it is difficult and wrong to dismiss providers’ anxieties about their safety. Healthcare workers are in the frontlines of this battle, and it is arguably a hospital’s responsibility to ensure that all providers feel safe in addition to being safe. Furthermore, the literature on modes of transmission is suggestive but not definitive, and estimates of the prevalence of asymptomatic infection are low but not zero. To the extent that a hospital’s supplies permit, allowing providers some discretion to use additional PPE may be the most practical course for the short term. Ensuring equitable treatment of all hospital employees, including both frontline and supporting staff members, is also important to maintain morale.

Over the long term, however, more evidence-based and measured infection control strategies are needed because SARS-CoV-2 is here to stay. We believe that several lines of evidence are needed to inform these strategies. First, we need better data on the transmission dynamics of SARS-CoV-2, in particular the degree to which infection is transmitted via the airborne route versus droplets and fomites during routine care. Second, a standardized and evidence-based list of aerosol-generating procedures must be defined, ideally based on studies documenting respiratory virus transmission rather than theoretical concerns alone. Third, we need more studies of the prevalence of positive tests in asymptomatic patients particularly in regions with high overall case rates. Ideally, these data will be gathered locally and repeatedly to allow providers and hospitals to titrate their infection control measures and PPE use for asymptomatic patients according to local risk. Fourth, a better understanding of the negative predictive value of both PCR and serological tests are needed to help optimize testing strategies. Fifth, studies should be conducted comparing the efficacy (or lack thereof) of N95 respirators compared to medical masks for preventing COVID-19 infections, similar to prior cluster randomized trials conducted for prevention of influenza in healthcare workers.^23,24^ Sixth, a better understanding of the significance of serological tests—in particular the degree and duration for which IgG antibodies confer immunity—may allow hospitals to strategically deploy healthcare workers to minimize risk as well as PPE use. Lastly, of course, the development and rapid deployment of an effective vaccine could completely change the infection control landscape. Until then, however, hospitals will continue to be driven by providers’ requests, peer hospitals’ actions, and professional societies’ pressure on the relentless march toward universal SARS-CoV-2 testing, universal masking of providers and patients, and universal use of face shields and N95 respirators for all patient encounters.

## References

[1] Verity R , Okell LC , Dorigatti I , et al. Estimates of the severity of coronavirus disease 2019: a model-based analysis. Lancet Infect Dis 2020. doi: 10.1016/S1473-3099(20)30243-7. PMC7158570 32240634

[2] Wu Z , McGoogan JM. Characteristics of and important lessons from the coronavirus disease 2019 (COVID-19) outbreak in China: summary of a report of 72,314 cases from the Chinese Center for Disease Control and Prevention. JAMA 2020;323:1239–1242. 10.1001/jama.2020.2648 32091533

[3] Infection prevention and control during health care when COVID-19 is suspected: Interim guidance. World Health Organization website. https://www.who.int/emergencies/diseases/novel-coronavirus-2019/technical-guidance/infection-prevention-and-control. Accessed May 8, 2020.

[4] Ong SWX , Tan YK , Chia PY , et al. Air, surface environmental, and personal protective equipment contamination by severe acute respiratory syndrome coronavirus 2 (SARS-CoV-2) from a symptomatic patient. JAMA 2020. Mar 4 [Epub ahead of print]. doi: 10.1001/jama.2020.3227. PMC7057172 32129805

[5] van Doremalen N , Bushmaker T , Morris DH , et al. Aerosol and surface stability of SARS-CoV-2 as compared with SARS-CoV-1. N Engl J Med 2020;382:1564–1567. 32182409 10.1056/NEJMc2004973 PMC7121658

[6] Santarpia JL , Rivera DN , Herrera V , et al. Transmission potential of SARS-CoV-2 in viral shedding observed at the University of Nebraska Medical Center. *MedRxiv* 2020 [Preprint]. doi: 10.1101/2020.03.23.20039446.

[7] Pan X , Chen D , Xia Y , et al. Asymptomatic cases in a family cluster with SARS-CoV-2 infection. Lancet Infect Dis 2020;20:410–411. 32087116 10.1016/S1473-3099(20)30114-6 PMC7158985

[8] Kimball A , Hatfield KM , Arons M , et al. Asymptomatic and presymptomatic SARS-CoV-2 infections in residents of a long-term care skilled nursing facility—King County, Washington, March 2020. Morb Mortal Wkly Rep 2020;69:377–381. 10.15585/mmwr.mm6913e1 PMC7119514 32240128

[9] Bai Y , Yao L , Wei T , et al. Presumed asymptomatic carrier transmission of COVID-19. JAMA 2020 Feb 21 [Epub ahead of print]. doi: 10.1001/jama.2020.2565. PMC7042844 32083643

[10] Hoehl S , Rabenau H , Berger A , et al. Evidence of SARS-CoV-2 infection in returning travelers from Wuhan, China. N Engl J Med 2020;382:1278–1280. 32069388 10.1056/NEJMc2001899 PMC7121749

[11] Ng OT , Marimuthu K , Chia PY , et al. SARS-CoV-2 infection among travelers returning from Wuhan, China. N Engl J Med 2020;382:1476–1478. 32163698 10.1056/NEJMc2003100 PMC7121487

[12] Gudbjartsson DF , Helgason A , Jonsson H , et al. Spread of SARS-CoV-2 in the Icelandic population. N Engl J Med 2020 Apr 14 [Epub ahead of print]. doi: 10.1056/NEJMoa2006100. PMC7175425 32289214

[13] Ai T , Yang Z , Hou H , et al. Correlation of chest CT and RT-PCR testing in coronavirus disease 2019 (COVID-19) in China: a report of 1,014 cases. Radiology 2020:200642. 10.1148/radiol.2020200642 PMC7233399 32101510

[14] Lee TH , Lin RJ , Lin RTP , et al. Testing for SARS-CoV-2: can we stop at two? Clin Infect Dis 2020. Apr 19 [Epub ahead of print]. doi: 10.1093/cid/ciaa459. PMC7188180 32306042

[15] Healthcare infection prevention and control FAQs for COVID-19. Centers for Disease Control and Prevention website. https://www.cdc.gov/coronavirus/2019-ncov/hcp/infection-control-faq.html. Updated April 19, 2020. Accessed April 23, 2020.

[16] Tran K , Cimon K , Severn M , Pessoa-Silva CL , Conly J. Aerosol-generating procedures and risk of transmission of acute respiratory infections to healthcare workers: a systematic review. PLoS One 2012;7:e35797. 22563403 10.1371/journal.pone.0035797 PMC3338532

[17] Aerosol generating procedures performed by interventional radiology: clinical notification from the Society of Interventional Radiology. Society of Interventional Radiology website. https://www.sirweb.org/practice-resources/covid-19-resources/covid-19-clinical-notification-3-26-20/. Published March 26, 2020. Accessed April 1, 2020.

[18] SOGH COVID-19 position statement - April 10th, 2020. Society of OB/GYN Hospitalists website. https://www.societyofobgynhospitalists.org/portfolio/education/. Published April 10, 2020. Accessed April 22, 2020.

[19] ASHA guidance to SLPs regarding aerosol-generating procedures - April 1, 2020. American Speech-Language-Hearing Association website. https://www.asha.org/SLP/healthcare/ASHA-Guidance-to-SLPs-Regarding-Aerosol-Generating-Procedures/. Published April 1, 2020. Accessed April 22, 2020.

[20] SAGES and EAES recommendations regarding surgical response to the COVID-19 crisis - March 29, 2020. Society of American Gastrointestinal and Endoscopic Surgeons website. https://www.sages.org/recommendations-surgical-response-covid-19/. Published March 29, 2020. Accessed April 22, 2020.

[21] Guo ZD , Wang ZY , Zhang SF , et al. Aerosol and surface distribution of severe acute respiratory syndrome coronavirus 2 in hospital wards, Wuhan, China, 2020. Emerg Infect Dis 2020;26(7). doi: 10.3201/eid2607.200885. PMC7323510 32275497

[22] Morgan DJ , Diekema DJ , Sepkowitz K , Perencevich EN. Adverse outcomes associated with contact precautions: a review of the literature. Am J Infect Control 2009;37:85–93. 19249637 10.1016/j.ajic.2008.04.257 PMC3557494

[23] Loeb M , Dafoe N , Mahony J , et al. Surgical mask vs N95 respirator for preventing influenza among healthcare workers: a randomized trial. JAMA 2009;302:1865–1871. 19797474 10.1001/jama.2009.1466

[24] Radonovich LJ Jr , Simberkoff MS , Bessesen MT , et al. N95 Respirators vs medical masks for preventing influenza among health care personnel: a randomized clinical trial. JAMA 2019;322:824–833. 31479137 10.1001/jama.2019.11645 PMC6724169

